# A novel oHSV-1 targeting telomerase reverse transcriptase-positive cancer cells via tumor-specific promoters regulating the expression of ICP4

**DOI:** 10.18632/oncotarget.3884

**Published:** 2015-05-06

**Authors:** Wen Zhang, Keli Ge, Qian Zhao, Xiufen Zhuang, Zhenling Deng, Lingling Liu, Jie Li, Yu Zhang, Ying Dong, Youhui Zhang, Shuren Zhang, Binlei Liu

**Affiliations:** ^1^ Department of Immunology, Cancer Institute & Hospital, Chinese Academy of Medical Sciences & Peking Union Medical College, Beijing 100021, China; ^2^ Department of Pathology, Cancer Institute & Hospital, Chinese Academy of Medical Sciences & Peking Union Medical College, Beijing 100021, China; ^3^ Hubei University of Technology, Nanhu, Wuchang District, Wuhan 430068, China

**Keywords:** oncolytic HSV-1, hTERT, tumor specific, ICP4, oncolytic virotherapy

## Abstract

Virotherapy is a promising strategy for cancer treatment. Using the human telomerase reverse transcriptase promoter, we developed a novel tumor-selective replication oncolytic HSV-1. Here we showed that oHSV1-hTERT virus was cytopathic in telomerase-positive cancer cell lines but not in telomerase-negative cell lines. In intra-venous injection in mice, oHSV1-hTERT was safer than its parental oHSV1-17+. In human blood cell transduction assays, both viruses transduced few blood cells and the transduction rate for oHSV1-hTERT was even less than that for its parental virus. *In vivo*, oHSV1-hTERT inhibited growth of tumors and prolong survival in telomerase-positive xenograft tumor models. Therefore, we concluded that this virus may be a safe and effective therapeutic agent for cancer treatment, warranting clinical trials in humans.

## INTRODUCTION

Oncolytic viruses, are a novel approach for cancer therapy, and have been intermittently tested for cancer treatment since the beginning of the 20th century [1–3], with the most extensive work having begun in the 1990's. Oncolytic viruses either naturally or are engineered so that they selectively replicate in cancer cells and induce cell lysis, which activates the host immune system against released tumor antigens [4]. A wide variety of oncolytic viruses are undergoing preclinical or clinical research, including viruses based on vaccinia, adenovirus, herpes simplex virus, reovirus, and Newcastle disease virus [5–8].

Oncolytic herpes simplex virus type-1 (oHSV-1) is one of the most promising cancer therapy agents [9–11]. A number of HSV-1 constructs have been designed to provide selective replication in tumor cells. One strategy involves the removal of the gene encoding ICP34.5, which is the major neuropathogenicity determinant providing interferon resistance [12]. The oncolytic virus which is most advanced in clinical development, talimogene laherparepvec, has the ICP34.5 and ICP47 encoding genes deleted, and expresses GM-CSF. This has been proved to be clinically effective in melanoma patients in a pivotal phase 3 trial [13]. Another strategy is to manipulate the HSV-1 genes that are critical for the control of replication [14], particularly genes that do not have homologues in host cells and whose function therefore cannot be complemented in the host. The essential immediate early protein ICP4 is essential for virus replication and could therefore be used to control replication in tumor cells if regulated by a tumor specific or selective promoter [15–17].

Recently, it has been shown that the human telomerase reverse transcriptase (hTERT) promoter could be a good candidate for the transcriptional control of expression of therapeutic genes in cancer gene therapy [20, 21]. Telomerase plays an important role in cancer cells [22], and contains three subunits, including a telomerase-associated protein [23], an RNA subunit [24] and the catalytic subunit (hTERT), which is the major determinant of telomerase activity [25]. Bioinformatics data indicate that hTERT is a susceptibility gene for the development of many cancers [26–28]. Telomerase activity and hTERT expression can be detected in 90% of cancer cells [28], but is usually absent in the normal somatic tissues or in benign tumors [29]. Therefore, utilizing the hTERT promoter to regulate the transcription of a virus key gene is a potential way to selectively target tumor cells by an oncolytic virus.

In this study, we therefore chose the hTERT promoter to drive the expression of ICP4 from the the HSV-1 genome. Previously, we used the 17+ strain to construct an oHSV-1, referred to as oHSV1-17+ in this manuscript, in which the ICP34.5 and ICP47 genes were removed [30]. Based on oHSV1-17+, we constructed a novel hTERT promoter-regulated oncolytic HSV-1 (oHSV1-hTERT), in which the ICP4 gene was controlled by the hTERT promoter core sequence. We tested the killing effects and selective replication of the oHSV1-hTERT virus in a broad array of cell lines with different telomerase activity. By driving the expression of ICP4 using the hTERT promoter, the tumors could be targeted with minimal toxicity to normal cells. Additionally, both oHSV1-hTERT and oHSV1-17+ demonstrated antitumor activity in xenograft models of hepatocellular carcinoma and gastric carcinoma, indicating that the oHSV1-hTERT virus to be of potential promise as a therapeutic agent for cancer.

## RESULTS

### Construction of an hTERT promoter-activated oHSV-1

**Figure 1:**
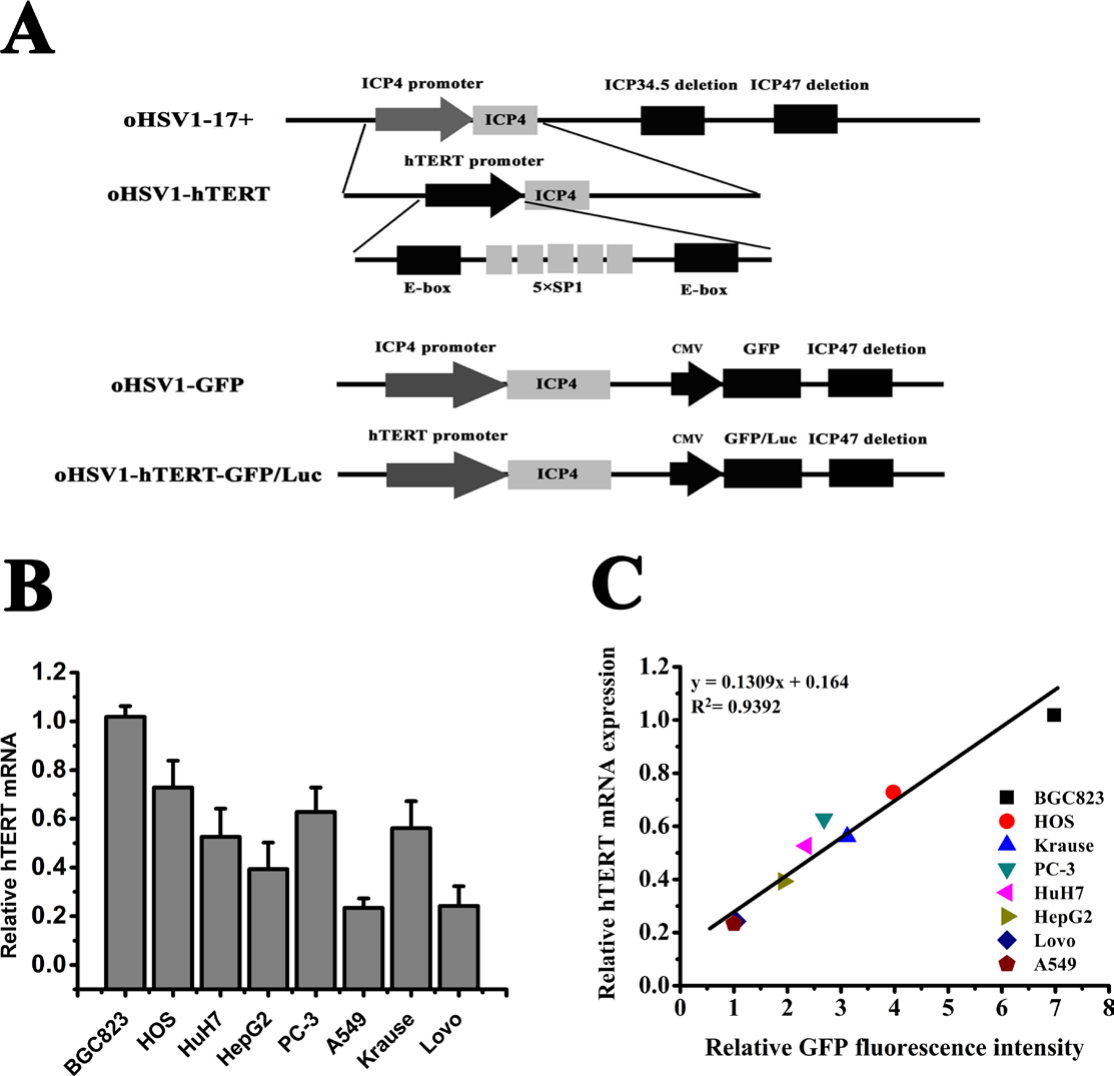
Replication of oHSV1-hTERT is correlated with telomerase activity **A.** Schematic construction of oHSV1-hTERT, oHSV1-GFP and oHSV1-hTERT-GFP/Luc. oHSV1-hTERT was developed from oHSV1-d34.5-d47, referred to as oHSV1-17+. The hTERT core promoter contains two E-box domains and five SP1 binding sites. The modifications included the deletion of the ICP47 and ICP34.5 genes, the replacement of the ICP4 promoter with the hTERT promoter and the insertion of the CMV promoter controlled GFP or luciferase expression cassette at the deleted ICP34.5 sites. **B.** Expression of TERT in different human cancer cell lines. The expression levels of TERT mRNA were measured using qRT-PCR and were normalized to the corresponding expression level of GAPDH. The bars represent the relative expression levels of mRNA. **C.** Relationship between TERT mRNA expression and GFP fluorescence intensity in human cancer cell lines. The expression levels of TERT mRNA were normalized to the level of BGC823, and the GFP fluorescence intensity was normalized to the level of A549.

oHSV1-hTERTp-ICP4 (oHSV1-hTERT), oHSV1-hTERTp-ICP4-CMVp-eGFP (oHSV1-hTERT-GFP) and oHSV1-hTERTp-ICP4-CMVp-Luc2 (oHSV1-hTERT-Luc), are shown in Fig. 1A. The endogenous ICP4 promoter of oHSV1-17+ was replaced with the hTERT core promoter, which contains five SP1 binding sites and two E-box domains. Additionally, the CMV promoter-driven expression cassette for green fluorescent protein (GFP) or luciferase (Luc) was inserted into the ICP34.5 site (Fig. 1A).

### Replication of oHSV1-hTERT is correlated with intracellular telomerase activity

To verify that the replication of the oHSV1-hTERT was regulated by the tumor-specific hTERT promoter and to evaluate the GFP expression profile, we first measured the hTERT mRNA expression of the cultured cell lines using qRT-PCR. The data showed that hTERT mRNA could be detected in the BGC823, HOS, PC-3, HepG2, HuH7, A549, Lovo, and Krause cells (Fig. 1B). However, hTERT mRNA was not detected in Saos-2 or Wi-38 cells. We then used oHSV1-hTERT at MOI = 0.1 to infect these cell lines and analyzed GFP fluorescence intensity using flow cytometry (Fig. 1C). The qRT-PCR data indicated that the intracellular transcription of hTERT widely varied, even in the hTERT positive cell lines. Because the GFP expression level would be expected to be associated with virus replication (in addition to any differential CMV promoter activity between the different cells), which was determined by ICP4 expression driven by the hTERT promoter, we hypothesized that the expression of GFP also correlated with the intracellular transcription of TERT. Indeed, the data clearly showed that GFP expression was mostly correlated to hTERT mRNA expression by curve fitting (R^2^ = 0.9392, Fig. 1C, Supplementary Fig. S1). This result suggested that the replication of the oHSV1-hTERT was determined by the intracellular transcription of TERT.

### oHSV1-hTERT specifically targeted the tumor cell lines with high telomerase activity *in vitro*

**Figure 2:**
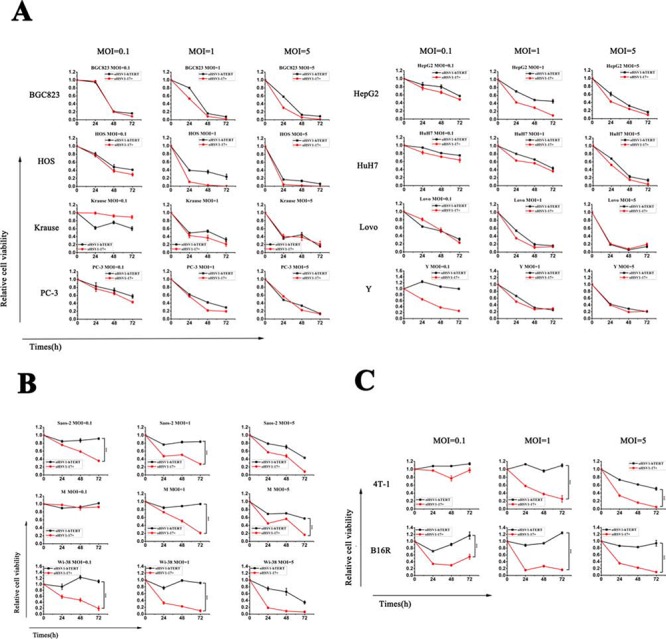
*In vitro* oncolytic activity comparison of oHSV1-hTERT and oHSV1-17+ **A.** oHSV1-hTERT and oHSV1-17+ were used to infect human cancer cell lines with high telomerase activity at the indicated MOIs for the indicated times. The human cancer cell lines included BGC823, HOS, Krause, PC-3, HepG2, HuH7, LoVo and Y. **B.** oHSV1-hTERT and oHSV1-17+ were used to infect the human cancer cell lines lacking telomerase activity at the indicated MOIs for the indicated times. The human cancer cell lines included Saos-2, Wi-38 and M. **C.** oHSV1-hTERT and oHSV1-17+ were used to infect mouse cancer cell lines at the indicated MOIs for the indicated times. The mouse cancer cell lines included 4T-1 and B16R. There was a significant difference in the oncolytic activity of oHSV1-hTERT and oHSV1-17+ (*p* = 0.0002, 0.0005, 0.0006, 0.0007 and <0.0001) for the Saos-2, Wi38, M, 4T-1 and B16R cells, respectively. Each value represents the mean ± SED of three independent samples.

To investigate the selective oncolytic activity of oHSV1-hTERT, the oHSV1-hTERT virus was compared to its parental virus (oHSV1-17+). Fig. 2A showed that oHSV1-hTERT exhibited a similar tumor cell killing ability as oHSV1-17+ in human tumor cell lines with positive TERT activity. All of the cell lines with positive TERT activity were very sensitive to oHSV1-hTERT or oHSV1-17+, with cell viability below 40% when infected with an MOI of 1. And except for Y cell line, there was no significant difference in the killing effect on positive TERT activity cell lines between oHSV1-hTERT and oHSV1-17+ at MOIs of 0.1. However, the human cell lines without detectable telomerase activity and the mouse cancer cell lines were still very sensitive to oHSV1-17+, but not to oHSV1-hTERT (Fig. 2B and 2C). In addition, oHSV1-hTERT exhibited cytotoxic activity, at MOI = 0.1 in most of the human tumor cell lines with high hTERT activity (Supplementary Fig. S2A). Three days after infection of the oHSV1-hTERT at an MOI of 1, viability of the individual cancer cell lines ranged from 7.6–39.61%. Interestingly, either the human cell lines with negative hTERT activity or the mouse cancer cell lines exhibited normal cell viability after infection with oHSV1-hTERT at an MOI of 1 or lower (Supplementary Fig. S2B and S2C). However, oHSV1-hTERT infection at an MOI of 5 could also induce irreversible toxicity in Saos-2, Wi-38, M and B16R cells. This toxicity at high MOI may have been due to high level of transient expression of non-ICP4 viral IE proteins, such as ICP0.

### oHSV1-hTERT viral replication is inhibited in telomerase-negative tumor cells

**Figure 3:**
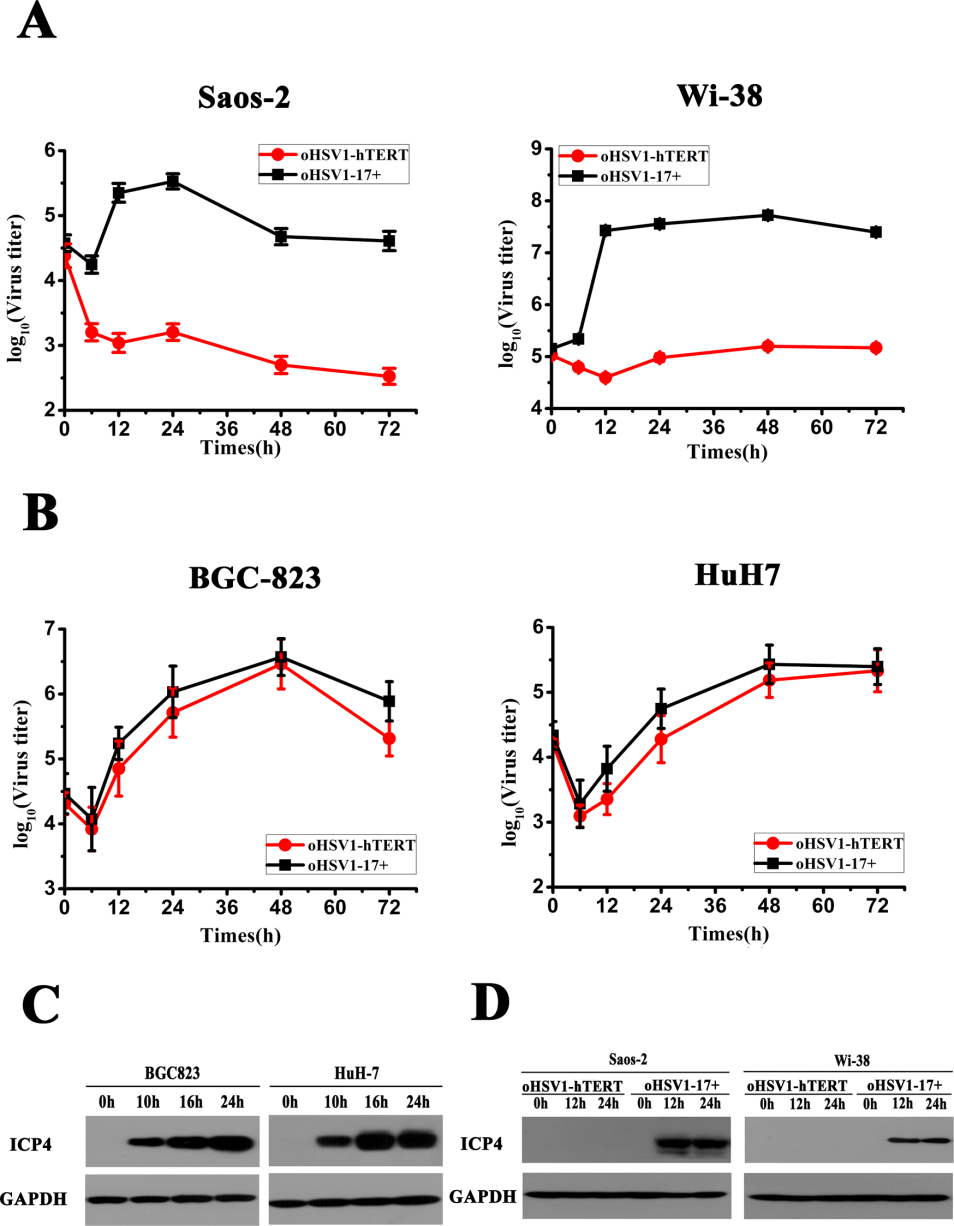
Comparison of oHSV1-hTERT and oHSV1-17+ replication **A.** oHSV1-17+ and oHSV1-hTERT replication were assessed in the telomerase-negative Saos-2 and Wi-38 cell lines by growth curve analysis at MOI = 0.1. **B.** oHSV1-17+ and oHSV1-hTERT replication were assessed in the telomerase-positive BGC823 and HuH7 cell lines by growth curve analysis at MOI = 0.1. **C.** The expression of ICP4 in the telomerase-positive cell lines was measured after oHSV1-hTERT infection at an MOI of 1 using western blot analysis. **D.** The expression of ICP4 in telomerase-negative cell lines was measured after oHSV1-hTERT or oHSV1-17+ infection at an MOI of 1 using western blot analysis.

To ensure that oHSV1-hTERT was unable to kill the telomerase negative tumor cells, we measured virus titers after infection with oHSV1-hTERT and oHSV1-17+. As shown in Fig. 3A, after approximately 6 h, the virus titers of oHSV1-17+ were markedly increased in both the Saos-2 and Wi-38 cells and, with cell lysis, oHSV1-17+ virus titers then began to fall. However, neither the Saos-2 and Wi-38 cells supported replication of oHSV1-hTERT. However, as shown in Fig. 3B oHSV1-hTERT exhibited a similar replicative capability as oHSV1-17+ in human tumor cell lines with positive TERT activity. Of note, similar results were obtained using western blot analysis. ICP4 was detected in the BGC823 and HuH7 cells infected with oHSV1-hTERT 10 h after infection (Fig. 3C). With a longer infection time, the expression of ICP4 increased. The expression of ICP4 protein was observed in the Saos-2 and Wi-38 cells infected with the oHSV1-17+ virus, but not in cells infected with the oHSV1-hTERT virus, until 24 h (Fig. 3D).

### oHSV1-hTERT induced necrosis, not apoptosis, in the cancer cells

**Figure 4:**
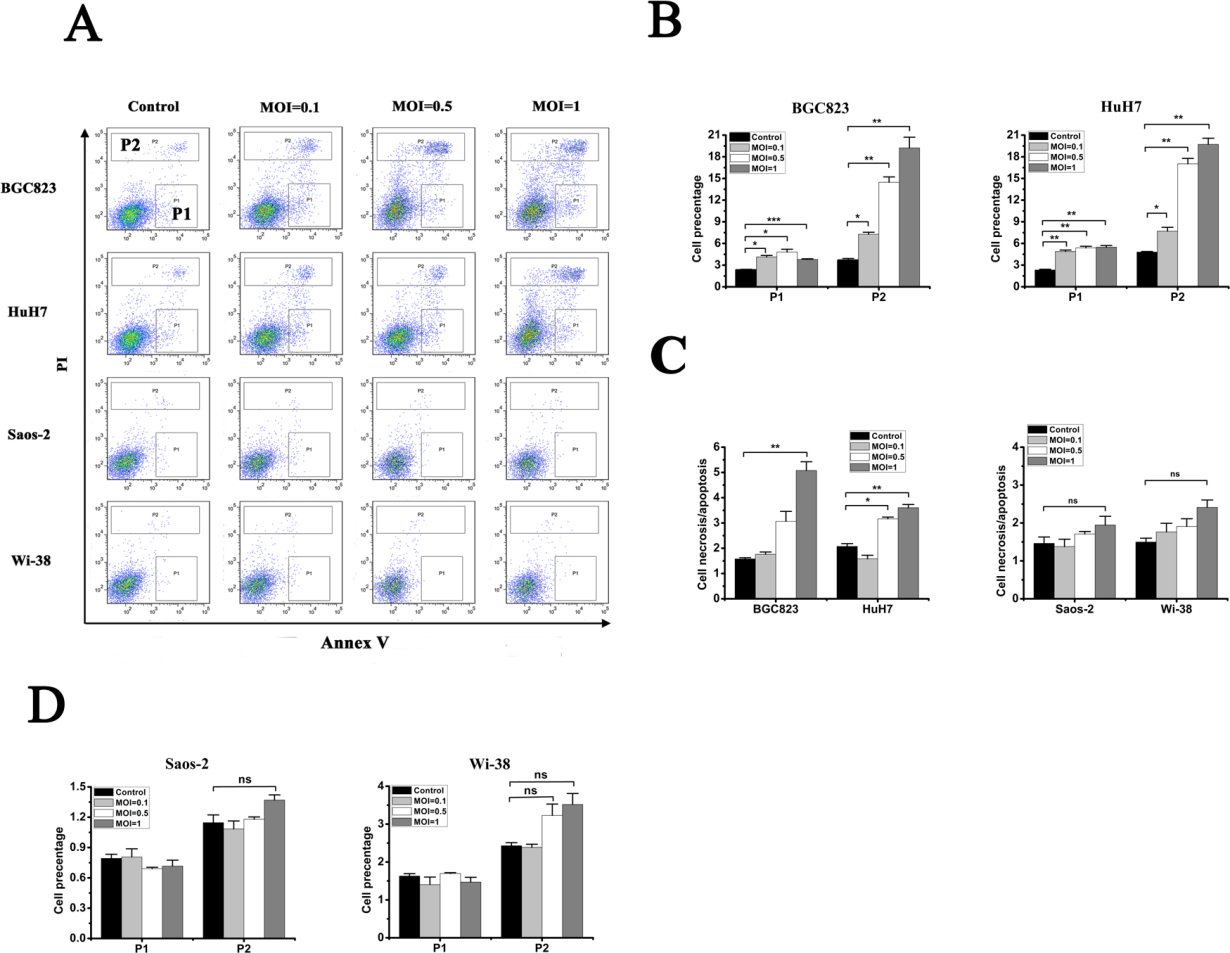
oHSV1-hTERT induces necrosis in telomerase-positive cancer cells **A.** Flow cytometry analysis of cancer cell lines after oHSV1-hTERT infection at the indicated MOIs. P1, apoptosis; P2, necrosis. **B.** The necrosis and apoptosis rates of the BGC823 and HuH7 were measured after oHSV1-hTERT infection. **C.** The necrosis/apoptosis rates were analyzed in both the telomerase-negative and telomerase-positive cell lines. necrosis/apoptosis = P2/P1. **D.** The necrosis and apoptosis rates of Saos-2 and Wi-38 were measured after oHSV1-hTERT infection. Each value represents the mean ± SED of three independent samples. *p < 0.05; **p < 0.01; ***p < 0.001.

Annexin-V/PI assays showed that oHSV1-hTERT induced necrosis in the telomerase activity positive tumor cells, but not apoptosis (Fig. 4A). DNA ladder assay also carried out to confirm the results (Supplementary Fig. S3). Both the proportion of necrotic and apoptosic BGC823 and HuH7 cells were statistically significantly different between the control group and the oHSV1-hTERT treatment group (Fig. 4B) and showed that oHSV1-hTERT primarily induced necrosis, not apoptosis (Fig. 4C). In contrast, the proportion of necrotic and the apoptosic Saos-2 and Wi-38 tumor cells showed no significant difference between the control group and the oHSV1-hTERT treatment group (Fig. 4C and 4D).

### oHSV1-hTERT is less toxic than oHSV1–17+

**Figure 5:**
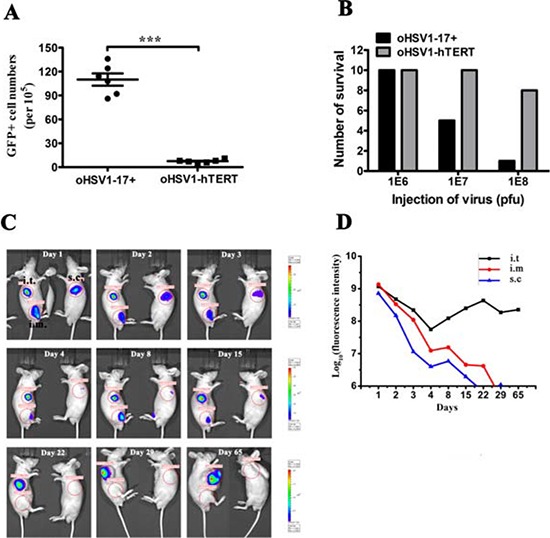
oHSV1-hTERT is tumor specific and safe **A.** The rates of GFP-positive cell in WBCs after oHSV1-hTERT-GFP or oHSV1-GFP infection. Each value represents the mean ± SED of three independent samples. ***p < 0.001. **B.** Acute toxicity test for oHSV1-17+ and oHSV1-hTERT. oHSV1-hTERT or oHSV1-17+ (1 × 10^6^, 1 × 10^7^ or 1 × 10^8^ pfu) was injected intraveinly. The number of mice survied was calculated two weeks after injection. **C.** Representative BGC823 mouse model for tracer analysis. oHSV1-hTERT-Luc (5 × 10^6^ pfu) was injected into the tumor (i.t.), muscle (i.m.) or subcutaneous tissue (s.c.) of the BGC823 mouse model (*n* = 3). Luciferase expression was measured using the IVIS Imaging System at the indicated times. **D.** The fluorescence intensity at different injection sites was measured at the indicated times. The BALB/c nude mice bearing BGC823 tumors were treated with oHSV1-hTERT-Luc.

Using flow cytometry, we measured the infection rate of white blood cells (WBCs) after exposure to virus. Peripheral blood samples were isolated from 6 healthy donors, and the data (Fig. 5A) showed that the number of WBCs transduced by oHSV1-GFP was significantly higher than that for oHSV1-hTERT-GFP (above 85 vs below 12 in 1 × 10^5^ cells and *p* < 0.0001), suggesting reduced replication for oHSV1-hTERT. In addition, in acute toxicity testing (Fig. 5B) no apparent toxicity was seen for both oHSV1-17+ and oHSV1-hTERT two weeks after 1 × 10^6^ pfu administration. However, with increasing dose, only 5 mice survived at 1 × 10^7^ pfu and 2 at 1 × 10^8^ pfu with oHSV1-17+ whereas no deaths occurred at 1 × 10^7^ pfu and 2 at 1 × 10^8^ pfu group for oHSV1-hTERT.

### oHSV1-hTERT replicated for an extended period in tumors *in vivo*

BGC823 cells were injected into BALB/c nude mice to induce the formation of subcutaneous tumors. When the tumor volume reached 5 mm × 5 mm, oHSV1-hTERT-Luc (5 × 10^6^ pfu) was injected intratumorally (i.t.), contralaterally subcutaneously (s.c.) or intramuscularly (i.m.) in 100 μl. We then observed the luciferase activity associated with oHSV1-hTERT-Luc replication for the next 65 days. As shown in Fig. 5C, the fluorescence quickly disappeared when the virus was injected at subcutaneous site. Interestingly, when injected intramuscularly, there was a slight increase in fluorescence on day 8, which became undetectable on day 22. Fluorescence was barely detected for the subcutaneous injection site on day 15. However, the intratumoral injection of oHSV1-hTERT-Luc decreased during the first 4 days, then increased and maintained a relatively stable level until day 65 (Fig. 5D). A similar result was also observed in a primary human neuroblastoma model (Supplementary Fig. S4). BGC823 model was repeated, with similar results being observed.

### Therapeutic effect of oHSV1-hTERT in subcutaneously xenografted BGC823 and HuH7 models

**Figure 6:**
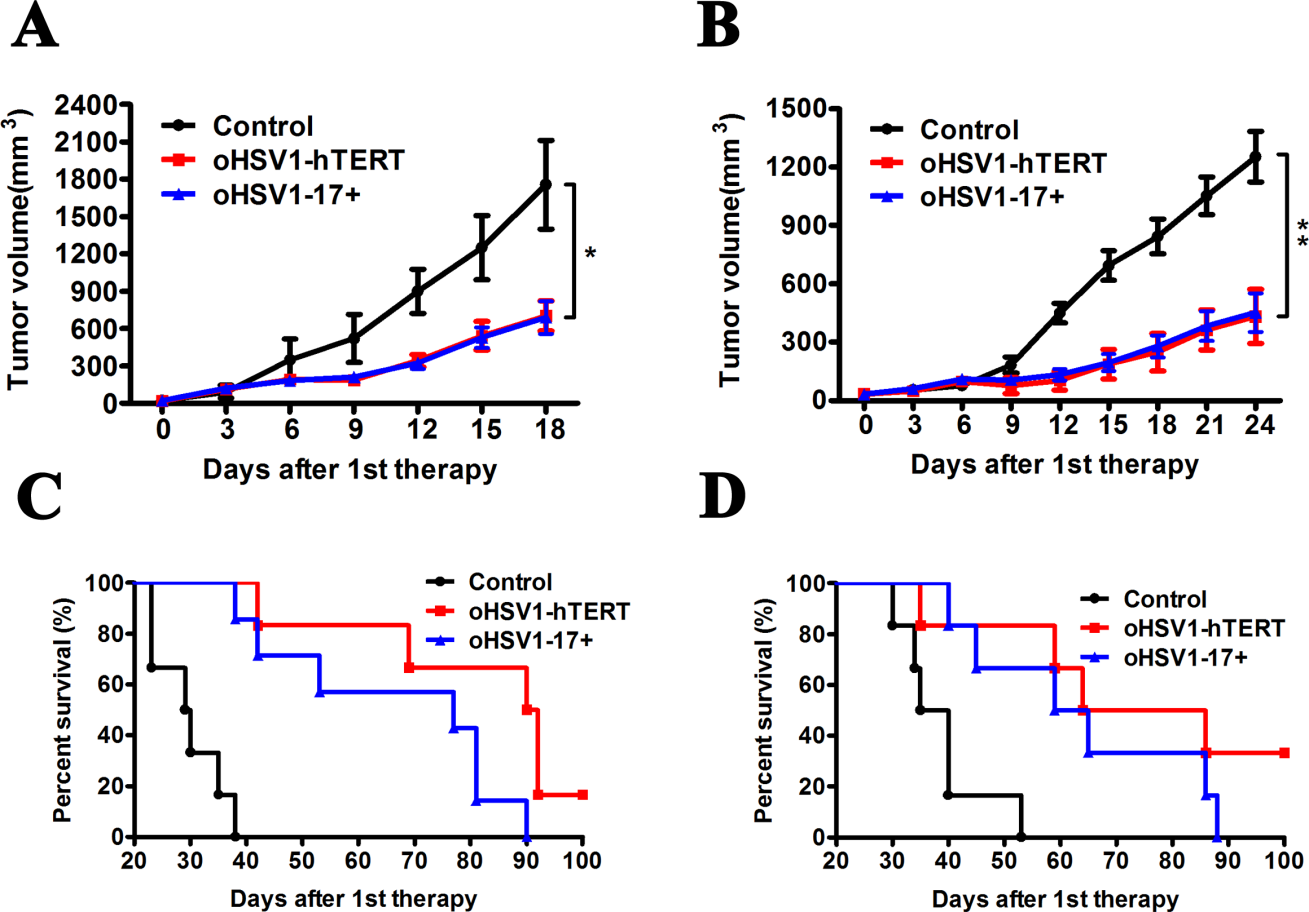
Therapeutic effect of oHSV1-hTERT *in vivo* **A.** The BGC823 average tumor volume within the different groups was measured every four days following treatments. The data represent the mean ± SEM (*n* = 6). *P* = 0.0432 and 0.0354 for oHSV1-hTERT and oHSV1-17+, respectively. **B.** The HuH7 average tumor volume within the different groups was measured every three days following treatments. The data represent the mean ± SEM (*n* = 6). *P* = 0.01 and 0.0086 for oHSV1-hTERT and oHSV1-17+, respectively. **C.** BGC823 model Kaplan–Meier survival curves (*n* = 6) for oHSV1-hTERT vs control. Median survival time: 29 days for control, 77 days for oHSV1-17+ and 91 days for oHSV1-hTERT. (*P* = 0.0004, 0.0005 for oHSV1-17+ and oHSV1-hTERT, respectively; log rank test) **D.** HuH7 model Kaplan–Meier survival curves (*n* = 6) for oHSV1-hTERT vs control. Median survival time: 38 days for control, 62 days for oHSV1-17+ and 75 days for oHSV1-hTERT. (*P* = 0.0082, 0.006 for oHSV1-17+ and oHSV1-hTERT, respectively; log rank test).

To evaluate the therapeutic effect of oHSV1-hTERT in telomerase positive tumors *in vivo*, we measured tumor growth and animal survival after treatment of the BGC823 and HuH7 xenografts by intratumoral injection of oHSV1-hTERT and oHSV1-17+. Virus was injected when tumors had reached 5 mm in diameter. Tumors were then observed for 18 days for BGC823 and 24 days for HuH7. Tumors reduced in volume for both the BGC823 and HuH7 tumors injected with both oHSV1-hTERT and oHSV1-17+ when compared to the control group (Fig. 6A and 6B), and the effects of oHSV1-hTERT were similar to those of oHSV1-17+. In additon, the mean survival times for the oHSV1-hTERT group and oHSV1-17+ group in both models were longer than those of the control group. The mean survival for the control mice was 29 days when injected with BGC823 cells and 38 days when injected with HuH7. In contrast, the mean survival for the oHSV1-hTERT treated mice was 91 days for the BGC823 and 75 days for the HuH7, and the mean survival for the oHSV1-17+ treated mice was 77 days for the BGC823 and 62 days for the HuH7 (Fig. 6C and 6D). There was no statistical difference between oHSV1-hTERT and oHSV1-17+ in mean survival. And a low telomerase xenografted mouse models, Saos-2 model, also showed that oHSV1-hTERT viral oncolytic activity was inhibited in telomerase-negative tumor cells (Supplementary Fig. S5).

## DISCUSSION

Viruses have been shown to be the most promising vectors for gene therapy [5, 6]. So far, many viruses have been modified to act as gene-transfer vectors to deliver a transgene of interest without undergoing replication [31]. On the other hand, destroying tumor cells might benefit from viral replication [32]. In our oncolytic strategy, described here, viral replication is selectively induced in tumor cells and not in normal cells.

A series of oHSV-1 viruses have been developed for solid tumors. Most of the oHSV-1 vectors have been engineered to delete the neurovirulence gene ICP34.5 to provide the property of selective replication in tumor cells, as well as reduce latent infection [12, 33–36, 30]. Other research has also been focused on the key genes needed for viral replication. ICP4 is the key activator of HSV-1 replication that leads to the synthesis of the early and late viral proteins [36–39]. Miyatake S et al [16] reported a virus which used the albumin promoter to regulate ICP4 expression. There have also been some other reports on the tumor-specific promoters used to regulate oncolytic HSVs. These include Wnt/T-cell factor controlled bM24-TE [18], calponin controlled d12.CALP [19], and hypoxia-inducible factor (HIF) responsive promoters driving oHSV1 replication [40]. Although oHSV constructs developed in these reports seem very attractive, there are some potential problems for tumor therapy. First, the promoters that have been used for regulating the oncolytic HSVs are also active in some normal cells, which resulted in some potential cytotoxic effects due to delayed viral replication. Second, these viruses are targeted to only a subgroup of specific tumor types [16–18]. Although HIF-HSV can be potentially used against a broader range of tumor types due to its overexpression in a wide variety of tumor cells, its antitumor effect can only be evaluated in hypoxic situations [41, 42].

In this study, we evaluated the feasibility of using an hTERT promoter to drive oncolytic HSV-1 replication to target tumor cells that have positive telomerase activity. Telomerase expression was demonstrated in most cancer types, but not in normal cells [28, 29]. oHSV1-hTERT was produced and replication was verified in BHK-ICP4 cells (Supplementary Fig. S6A), as the virus can only replicate in cells that stably express the ICP4 protein (Supplementary Fig. S6B). Our data showed that oHSV1-hTERT replication was regulated by intracellular TERT transcription. Even in a cell with a relatively low telomerase activity, the hTERT promoter could still trigger ICP4 expression, resulting in the replication of the virus.

As previously reported, oHSV-1 that lacks ICP34.5 and ICP47 infects most tumor cells types, replicates rapidly in the infected cells and has been used for solid tumor therapy. In our study, we also compared the oncolytic activity of oHSV1-hTERT and its parental vector oHSV1-17+. The data showed that oHSV1-hTERT had generally similar oncolytic activity to that of oHSV1-17+ on telomerase positive cancer cell lines. Surprisingly, even though we have replaced the promoter for ICP4, there appears to be no negative effect on the oncolytic activity in cancer cells lines with telomerase activity *in vitro*. Moreover, oHSV1-hTERT was tumor specific and failed to lyse telomerase activity-negative cell lines. Some studies have shown that HSV infection of tumor cells and normal cells can induce the expression of hTERT mRNA, resulting in hTERT promoter activity [43, 44]. We also compared the expression of hTERT mRNA after infection by oHSV1-hTERT and oHSV1-17+, and our data indicated oHSV1-hTERT infection failed to increase the level of hTERT mRNA (Supplementary Fig. S7A, Supplementary Table S1). These results suggest that oHSV1-hTERT is a tumor specific oncolytic agent.

An ideal oncolytic virus agent should have very few side effects. In our study, even in the immunodeficient xenograft tumor models, oHSV1-hTERT replication was restricted to the tumor, without spread into the surrounding nomal tissues. Moreover, we demonstrated that oHSV1-hTERT is less toxic than its parental oHSV1-17+, and the latter virus appeared to have a higher infection rate in normal human blood cells.

In conclusion, we have demonstrated that oHSV1-hTERT replication can be strictly controlled by the regulation of ICP4 expression by the hTERT promoter. Our data suggest that oHSV1-hTERT is a potent oncolytic virus and that testing for the treatment of tumors with positive telomerase activity is warranted.

## MATERIALS AND METHODS

### Cell lines and cell culture

The tumor cell lines, including LoVo (human colorectal cancer cell), PC-3 (human prostate cancer cell), HepG2 (human hepatocarcinoma cell), Krause (human renal carcinoma), Saos-2 (human osteosarcoma cell), Wi-38 (normal human fetal lung fibroblast cell), BGC823 (human gastric cancer cell), HuH7 (human hepatocarcinoma cell), BHK-ICP4 (baby hamster syrian kindey cell), Vero (kidney epithelial cells), 4T1 (mouse mammary carcinoma cell), B16 (mouse melanoma cell), B16R (mouse melanoma cell), M and Y (human bronchial epithelial cell), were used in this study. See Supplementary Methods for the details of cell information and culture condition.

### Plasmid construction

See Supplementary Methods.

### Virus construction

The oHSV1-hTERTp-ICP4 (oHSV1-hTERT) virus with the endogenous ICP4 promoter replaced with the hTERT promoter was derived from oHSV1-17+ [30]. See Supplementary Fig. S8.

### Quantitative analysis of hTERT mRNA levels by one-step real time RT-PCR

Total RNA was extracted from cultured cells using the TRIZOL reagent (Invitrogen, USA), according to the manufacturer's instructions. The cDNA library was then reverse transcribed using ReverTra Ace 1PCR RT Master Mix kit (Toyobo, Japan) according to the manufacturer's instructions. Quantitative RT-PCR (qRT-PCR) was performed using SYBR Green I Mix kit (Toyobo, Japan) with a 7300 Real-Time PCR System (ABI, USA) as described. The specific primers are listed in Supplementary Methods.

### CCK8 cell viability assay and viral growth curves

Cell viability was measured using a CCK-8 assay by Cell Counting kit-8 (DOJINDO, Japan). See Supplementary Methods.

For viral growth curves, the testing cells were seeded in 6-well plates and were infected with oHSV1-hTERT or oHSV1-17+ at an MOI of 0.1. The cell plates were gathered and kept in a −80°C refrigerator at 0, 6, 12, 24, 48, and 72 h. The virus titer of each time point was determined using the plaque-forming units (pfu) method.

### Western blot analysis of ICP4 expression

The ICP4 protein levels were assessed using standard western blot techniques; western blots were performed using the HSV-1 ICP4 (sc-69809, Santa Cruz, USA) and GAPDH primary antibodies (E021010-03, EarthOx, USA) and the HRP-conjugated goat anti-mouse IgG (H+L) secondary antibody (E030110-01, EarthOx, USA).

### Flow cytometry analysis

Cells were seeded in 6-well plates. After treatment with virus at an MOI of 0.1 for 48 h, the cells were harvested and analyzed by flow cytometry for GFP fluorescence intensity. For cell apoptosis analysis, the cells were seeded in 6-well plates at a density of 5 × 10^5^ cells/well; then, every cell line was infected with virus at an MOI of 1 for 24 h. Cell apoptosis was measured using the Dead Cell Apoptosis Kit (Invitrogen, USA), according to the manufacturer's instructions.

### Blood sample preparation

4 ml peripheral blood samples were prepared with heparinized tubes and incubated with lysis buffer (NH_4_Cl, 0.15 M; EDTA, 0.1 mM; KHCO_3_, 10 mM; PH = 7.2) at RT for 5 min. After centrifugation, the supernatant was discarded and the cell pellets were washed for twice. Following centrifugation, the cells were resuspended, infected with oHSV1-hTERT-GFP at pfu = 5 × 10^6^, and at 37°C in a humidified atmosphere of 5% CO_2_ for another 24 hours. Finally, the cells were collected and analysed with flow cytometry.

### Acute toxicity test

60 BALB/c mice, aged 6 to 8 weeks, were purchased from the Animal Center of the Chinese Academy of Medical Sciences, Beijing, China. The mice were randomly divided into six groups, 10 per group. The mice in each group were intra-tailveinly injected once of one dose of oHSV1-17+ or oHSV1-hTERT (1 × 10^6^, 1 × 10^7^ or 1 × 10^8^ pfu). The number of mice survival were counted two weeks after virus administration.

### Animal experiment

BALB/c nude mice, aged 6 to 8 weeks, were purchased from the Animal Center of the Chinese Academy of Medical Sciences, Beijing, China. HuH7 (1 × 10^6^) or BGC823 (5 × 10^5^) or Saos-2 (1 × 10^6^) cells were injected into the right flanks of the mice. At the time of tumor induction, the mice were randomly divided into three groups, the oHSV1-hTERT group, the oHSV1-17+ group and the control group. Once tumors had reached 5 mm in diameter, the mice were given intra- or peri-tumoral injection of 5 × 10^6^ pfu of virus in a volume of 100 μl once every three days for three injections in total. In the control, no virus, group, the mice received injections of the same volume of DMEM/F12 medium. Tumor sizes were measured using calipers every 3 days for HuH7 and BGC823, and 5 days for Saos-2. Tumor volumes were estimated using the following formula: a × b^2^ × 0.5, in which a and b represent the maximal and minimal diameters, respectively. In the experiments, the mice were euthanized by cervical dislocation when tumor volumes reached 2000 mm^3^ to avoid unnecessary suffering.

Viruses were also constructed expressing luciferase (Luc) for these studies (Fig. 1A). To examine the replication of oHSV1-hTERT mice, 5 × 10^6^ pfu of the oHSV1-hTERT-Luc virus was injected in 100 μl once into the tumor, muscle and subcutaneous tissue of the same mice. Fluorescence was examined using the IVIS Imaging System (Caliper Life Sciences, Hanover, Germany) at day 1, 2, 3, 4, 8, 15, 22, 29, and 65.

### Statistical analysis

All quantitative data are reported as the mean ± SED. Statistical analysis was performed for multiple comparisons using an analysis of variance test and Student's *t*-test. A *P* value < 0.05 was considered statistically significant.

## SUPPLEMENTARY DATA


