# Supplementary material for: A novel oHSV-1 targeting telomerase reverse transcriptase-positive cancer cells via tumor-specific promoters regulating the expression of ICP4
Source: Oncotarget. 2015 May 6;6(24):20345–55. doi: 10.18632/oncotarget.3884 (PMC4653009; doi:10.18632/oncotarget.3884)
Supplement: Supplementary file 1 [file oncotarget-06-20345-s001.pdf]

SUPPLEMENTARY DATA

Cell culture

The tumor cell lines, including LoVo, PC-3, HepG2, Vero, 4T1, B16, were purchased from ATCC and maintained in our laboratory. Krause, U2OS, Saos-2 and Wi-38 were purchased from the Cell Resource Center (IBMS, CAMS/PUMC). BGC823 cell line was a gift from BGI-Shenzhen (Shenzhen, China). The HuH7 cell line was a gift from Dr. Yuming Guo. The M and Y cell lines were a gift from Professor Kaitai Zhang (Cancer Hospital/Institute, CAMS, Beijing). Both the M and Y cell lines were noncancerous bronchial epithelial cells transformed with the SV40 virus. The Y cell line possesses high telomerase activity, whereas the M cell line lacks telomerase activity, as measured by gene chip. The BHK-ICP4 cell line, which is BHK-21 cells that stably express ICP4, was constructed by our laboratory. And the B16R cell line, which was stably transfected with an HSV receptor, was also constructed by our laboratory.

The tumor cell lines, including BGC823, LoVo, PC-3, HepG2, U2OS, Vero, 4T1, B16, were cultured in DMEM/F12 medium supplemented with 10% fetal bovine serum (FBS). The Saos-2 cells were cultured in McCoy's 5A medium supplemented with 10% FBS, and the Wi-38 cells were cultured in MEM medium supplemented with 1% NEAA and 10% FBS. All cell lines mentioned above were incubated at 37°C in a humidified atmosphere of 5% CO_2.

Plasmid construction

A number of plasmids were constructed to replace the virus's ICP4 promoter with the tumor-specific hTERT promoter. To construct the pICP4del plasmid, the ICP4 upstream (US) and downstream (DS) flanking regions (FLRs) were amplified using the ICP4USf/ICP4USr (CCCTCCAGACGCACCGGAGTCGGGGG/ AAGTCGACTCTAGAGGATCGATCTCTGACCTGA GATTGGCGGCACTGAGGTA) and ICP4DSf/ICP4DSr primer pairs (AAAAGTCGACCTGCAGGCATGCT AACGAGGAACGGGCAGGGGGC/AAAAAAGCTT GCATGCCCACGTGCGCGGGGCCAGACGGGCT), respectively. The ICP4 US FLR was cut with SalI, and the DS FLR was cut with SalI/HindIII; the two fragments were mixed and ligated to the pSP73 plasmid (Promega, USA) that was digested with SalI and HindIII to create the pICP4del plasmid, which was verified by sequencing. The pICP4del-eGFP plasmid was derived from the pICP4del plasmid by inserting a CMV-GFP cassette into its EcoRV site. The CMV-GFP cassette was cut from the pcDNA3.1-EGFP plasmid (YRGENE, Beijing) with EcoRI and XhoI and was blunt-ended.

To construct pICP4del-hTERTp-ICP4, we first synthesized the hTERT core promoter and inserted it into the pUC57 plasmid to generate the phTERTp plasmid. The hTERT core promoter was cut out from the phTERTp plasmid by NruI and HindIII digestion and was inserted into the pcDNA3-NHN plasmid that was pre-cut with NruI and HindIII to generate the pcDNA3-NHN-hTERTp plasmid. The pCDNA3-NHN plasmid was constructed by inserting the self-complement linker (GCTAGCGTTAACGCTAGC) into pcDNA3 that was pre-cut with PvuII. The ICP4 gene was amplified in three overlapping fragments: ICP4^{1st}, ICP4^{2nd} and ICP4^{3rd}. These fragments were separately cloned into pSP73 to give rise to pSP73-ICP4^{1st}, pSP73-ICP4^{2nd} and pSP73-ICP4^{3rd}, which were verified by sequencing. The primers used to amplify the ICP4 fragments are listed in Supplementary Table S1. Then, the three ICP4 fragments were cut out of their respective plasmids using the EcoRI and BsrGI, BsrGI and PvuI and PvuI and XhoI restriction enzymes, respectively. These fragments were mixed and ligated to the pcDNA3-NHN plasmid that was digested with EcoRI and XhoI to generate pcDNA3-NHN-hTERTp-ICP4. Finally, the hTERT-ICP4 fragment was digested from pcDNA3-NHN-hTERTp-ICP4 using PmeI and HpaI and was inserted into pICP4del to generate pICP4del-hTERTp-ICP4. Additionally, to make an ICP4 complementary cell line, the pcDNA3-CMV-ICP4 plasmid was created using the following steps. The ICP4 gene was released by EcoRI and XhoI digestion from pcDNA3-NHN-hTERTp-ICP4 and was ligated into the pcDNA3 plasmid that was digested with EcoRI and XhoI to generate pcDNA3-CMV-ICP4.

Two other shuttle plasmids were constructed to insert the eGFP and luciferase genes into the ICP34.5 site of the virus genome. The pdICP34.5 plasmid was previously described (26), whereas the pGL4.17-CMVp-Luc2/Neo plasmid was kindly provided by Dr. Chen Ling (Cancer Hospital/Institute, CAMS, Beijing). The GFP and luciferase expression cassettes from pcDNA3.1-EGFP and pGL4.17_CMVp_Luc2/Neo were removed through EcoRV and BamHI digestion and were blunt ended with T4 DNA polymerase; these cassettes were then cloned into the AfeI site of pdICP34.5 to generate pd34.5-CMVp-eGFP and pd34.5-CMVp-Luc2, respectively. These two plasmids were then verified by sequencing.

Virus construction

New viral vector was derived from oHSV1-d34.5-d47, which is referred to as oHSV1-17+, was previously described (44).

The pICP4del-eGFP and pICP4del-hTERTp-ICP4 vectors were used to construct oHSV1-hTERT, a descendent of oHSV1-17+, through two rounds of homologous recombination. Subsequently, the oHSV1-hTERT vector was further modified by the insertion of either the eGFP or luciferase (Luc2) expression cassette from pd34.5-CMVp-eGFP or pd34.5-CMVp-Luc2 inserted into the ICP34.5 site, giving rise to oHSV1-hTERTp-ICP4-CMVp-eGFP (oHSV1-hTERT-GFP) and oHSV1-hTERTp-ICP4-CMVp-Luc2 (oHSV1-hTERT-Luc), respectively.

The oHSV1-17+ stocks were prepared by infecting Vero cells and the oHSV1-hTERT based vectors were prepared by infecting BHK-ICP4 cells with 0.01 plaque-forming units (pfu)/cell. BHK-ICP4 is a complementary cell line for ICP4-deficient viruses. This cell line was made by transfecting the BHK-21 cells with the pcDNA3-CMV-ICP4 plasmid. The viruses were harvested 72 hours after infection, were freeze/thawed once and were purified by centrifugation at 3,000 rpm to remove cell debris, followed by a high speed centrifugation at 12,000 rpm to pellet the virus. The purified viruses were dissolved in SFM, titrated, divided into aliquots, and stored at −80°C until use.

CCK8 cell viability assay

The cells were seeded at a concentration range from 2×10^4 to 2×10^5 cells/ml and were seeded in 96-well plates at 100 µL per well. Each sample was run in triplicate. To test the effect of multiplicity of infection (MOI) on cell viability, every cell line was infected 24 h later with oHSV1-hTERT or oHSV1-17+ at MOIs of 0.1, 1 and 5. After culture for 24, 48 and 72 h, the culture medium was removed from the plates, and 100 µl of a mixture containing 10% CCK8 was added and incubated for an additional 4 h. The plates were examined using a model 550 microplate reader (BIO-RAD, Japan) at 450 nm with a reference of 655 nm.

DNA ladder

The cells and culture were harvested and lysed in the buffer. After Proteinase K treatment, DNA was gathered and washed by 75% ethanol. And the DNA ladder was analysed by 2% agarose gel electrophoresis.

Quantitative RT-PCR primers

hTERT, forward primer, 5′-CCGATTGTGAACATGGACTACG-3′ and reverse primer, 5′-CACGCTGAACAGTGCCTTC-3′; GAPDH, forward primer, 5′-TGTGGGCATCAATGGATTTGG-3′ and reverse primer, 5′-ACACCATGTATTCCGGGTCAAT-3′.

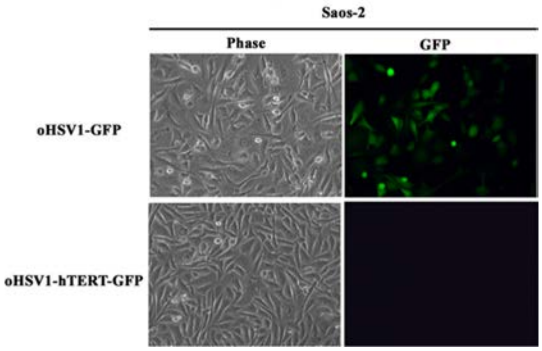

Supplementary Figure S1: Selective replication of oHSV1-hTERT-GFP in Saos-2. Images of Saos-2 cells were recorded for 24 h after oHSV1-hTERT-GFP or oHSV1-GFP infection at an MOI of 1. Selected images taken at the indicated time points show cell morphology by phase-contrast microscopy (left) and GFP expression under fluorescence microscopy (right). Original magnification, ×200.

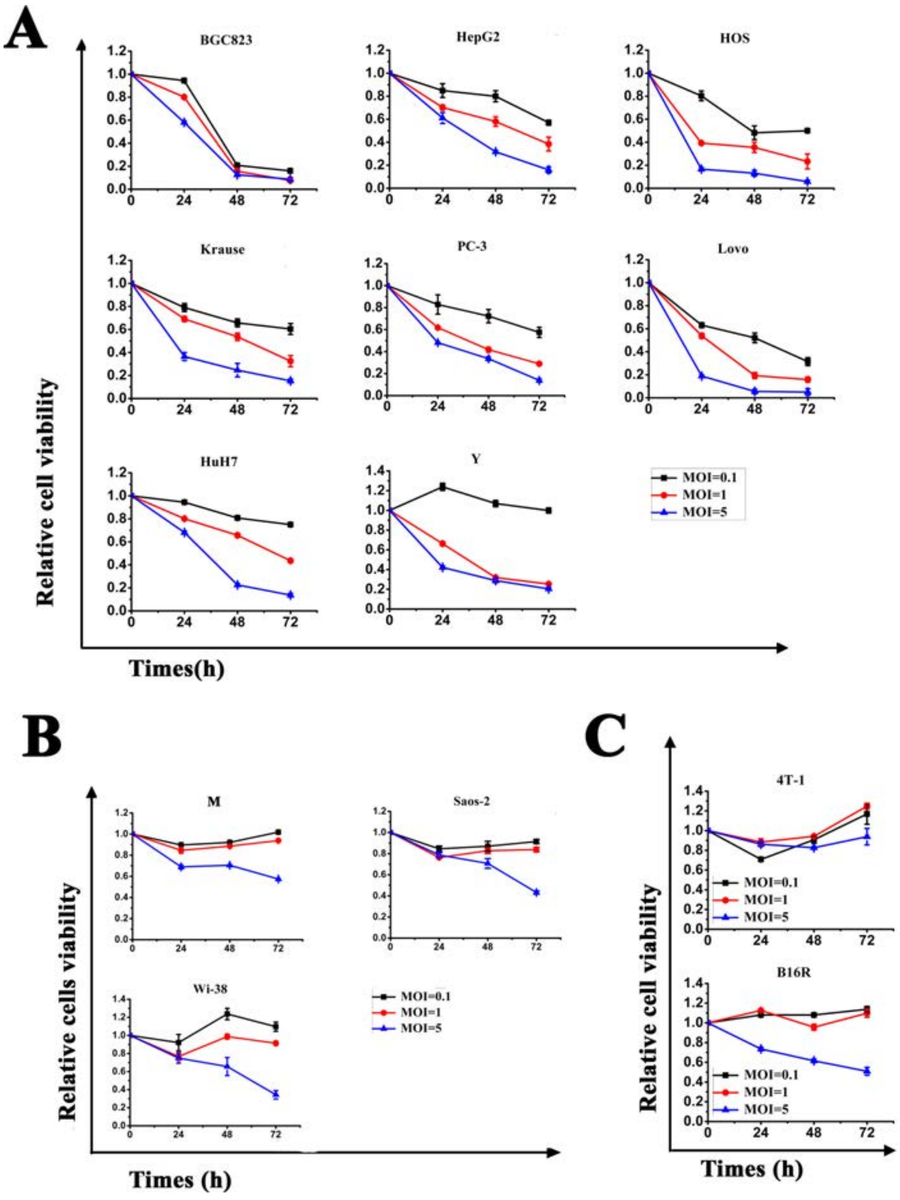

Supplementary Figure S2: *In vitro* oncolytic activity of oHSV1-hTERT. A. oHSV1-hTERT was used to infect human cancer cell lines with high telomerase activity at the indicated MOIs for the indicated times. The human cancer cell lines included BGC823, HOS, Krause, PC-3, HepG2, HuH7, LoVo and Y (the cell viability was 7.6% for BGC823, 38.39% for HepG2, 23.29% for HOS, 43.66% for HuH7, 32.49% for Krause, 15.83% for LoVo, 28.96% for PC-3, and 25.24% for Y, respectively). **B.** oHSV1-hTERT was used to infect the human cancer cell lines lacking telomerase activity at the indicated MOIs for the indicated times. The human cancer cell lines included Saos-2, Wi-38 and M. **C.** oHSV1-hTERT was used to infect mouse cancer cell lines at the indicated MOIs for the indicated times. The mouse cancer cell lines included 4T-1 and B16R. Each value represents the mean ± SED of three independent samples.

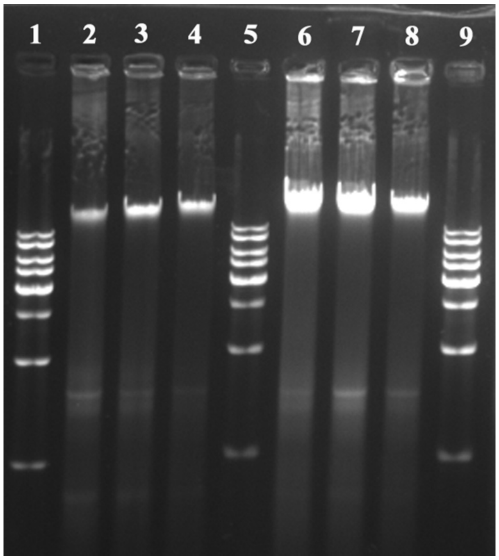

Supplementary Figure S3: DNA ladder analysis. Lane 1, 5, and 8: Marker; Lane 2: BGC823; Lane 3: BGC823+oHSV1-hTERT; Lane 4: BGC823+oHSV1-17+; Lane 6: HuH-7; Lane 7: HuH-7+oHSV1-hTERT; Lane 8: HuH-7+oHSV1-17+.

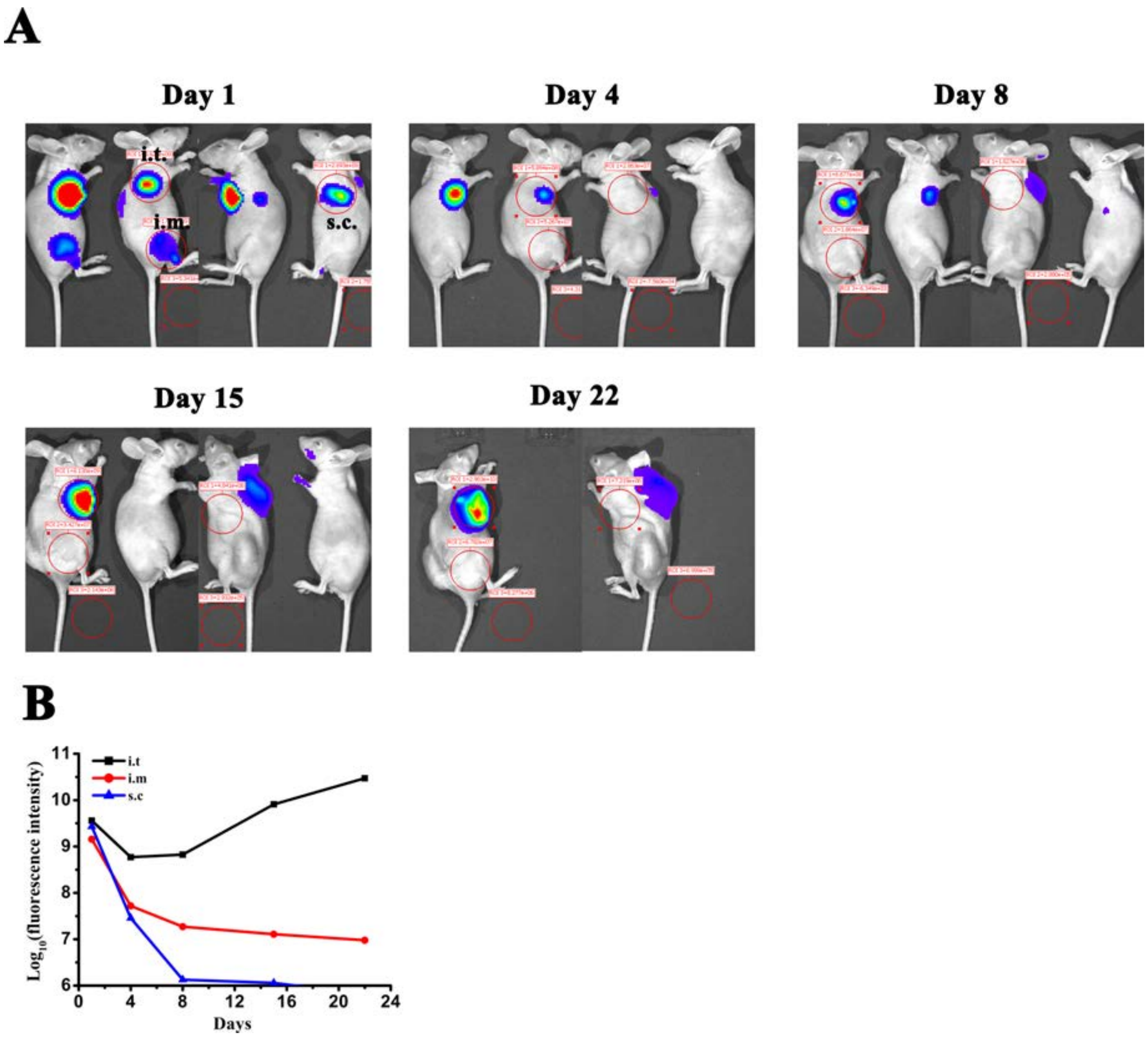

Supplementary Figure S4: Selective replication of oHSV1-hTERT in xenografted primary human neuroblastoma cells. A. oHSV1-hTERT-Luc (5 × 10^6 pfu) was injected into the tumor, muscle or subcutaneous tissue of the xenografted mouse model. Luciferase expression was measured using the IVIS Imaging System at the indicated times. **B.** The fluorescence intensity at different injection sites was measured at the indicated times.

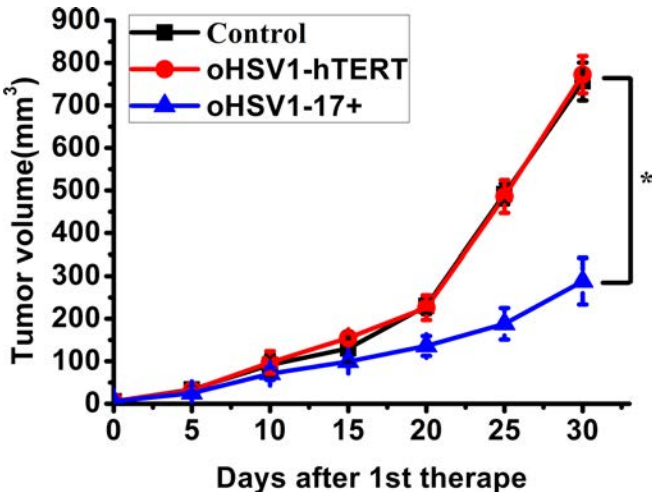

Supplementary Figure S5: Therapeutic effect of oHSV1-hTERT in Saos-2 xenografted model. A. The Saos-2 average tumor volume for all groups was measured every three days following treatments. The data represent the mean ± SEM (*n* = 6). *P* = 0.0441 and 0.0454 for oHSV1-hTERT and control, respectively.

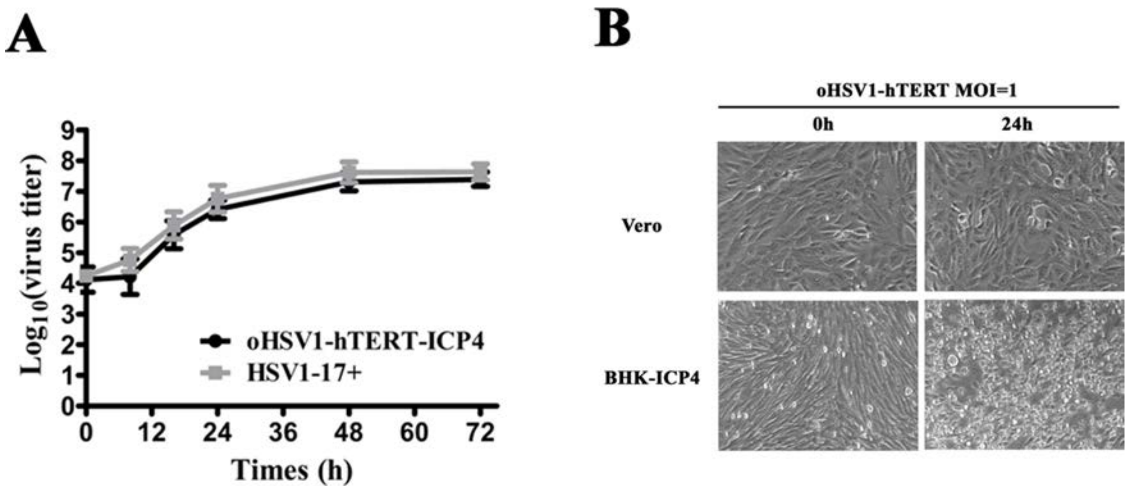

Supplementary Figure S6: A. oHSV1-17+ and oHSV1-hTERT replication were assessed in BHK-ICP4 cell lines by growth curve analysis with MOI = 0.1. B. oHSV1-hTERT was used to infect Vero and BHK-ICP4 cells at the indicated MOIs for the indicated times.

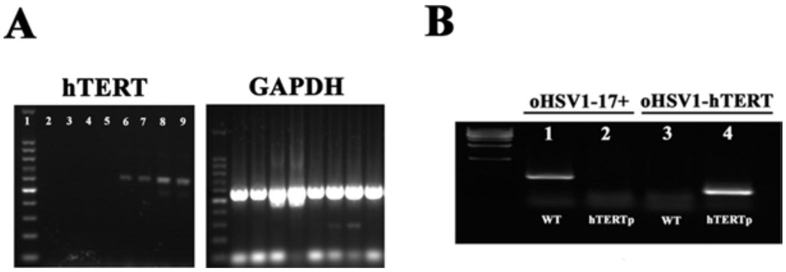

Supplementary Figure S7: A. Level of TERT mRNA in different human cancer cell lines after oHSV1-hTERT infection. The level of TERT mRNA was shown on the left, and GAPDH was shown on the right. Lane 1: Marker; Lane 2: Wi-38; Lane 3: Wi-38 + oHSV1-hTERT; Lane 4: Saos-2; Lane 5: Saos-2 + oHSV1-hTERT; Lane 6: HuH-7; Lane 7: HuH-7 + oHSV1-hTERT; Lane 8: BGC823; Lane 9: BGC823 + oHSV1-hTERT. **B.** The PCR for ICP4 promoter was analyzed by agarose gel electrophoresis. Line 1 and 3 were amplified with the primers from ICP4 wild type promoter, and Line 2 and 4 were amplified with the primer from hTERT promoter.

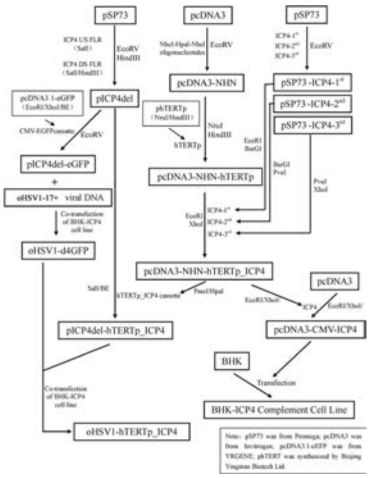

Supplementary Figure S8: The procedure of oHSV1-hTERT construction.

Supplementary Table S1. The primers of ICP4, ICP4 promoter and hTERT promoter

Primer name	Sequence
ICP4-1st sence	ttttttgaattc^{147105}atggcgtcggagaacaagcagcgcc^{147129}
ICP4-1st antisence	^{148279}tggagccaccccatggcctccgcgt^{148255}
ICP4-2st sence	^{148205}cgacgccgcgcagcagtacgccctg^{148229}
ICP4-2st antisence	^{149739}cggcgggggcgggcccggcgcaccg^{149715}
ICP4-3st sence	^{149675}cctcatgtttgacccgcgggccctg^{149699}
ICP4-3st antisence	ttttttctcgag^{151001}ttacagcaccccgtccccctcgaac^{150977}
ICP4 wild type promoter sence	ggactatatgagcccgaggacgccccgatcgtccacacg
ICP4 wild type promoter antisence	aaaggacagggacggccgatccccctcccg
hTERT promoter sence	tggcccctccctcgggttaccccacagcctaggccgatt
hTERT promoter antisence	ttcccacgtgcgcagcaggacgcagcgctg
